# Electrochemical Determination of the Prostate Anti-Cancer Drug Nilutamide Using Gold Nanoparticle–Polymer Based Electrode

**DOI:** 10.1021/acsomega.6c01799

**Published:** 2026-04-25

**Authors:** B. Büşra Karakaş, Z. Yaren Şahin, Aydan Elçi, Şükriye Karabiberoglu, Zekerya Dursun

**Affiliations:** Faculty of Science, Department of Chemistry, 37509 Ege University, 35100 Bornova, İzmir, Turkey

## Abstract

Gold nanoparticles decorated poly­(thiophene) modified glassy carbon electrode (Au/pTh/GCE) was used for the determination of nilutamide in pharmaceutical tablet. The electrochemical sensor was prepared by cyclic voltammetric technique on a glassy carbon electrode (GCE) modified with poly thiophene (pTh) and gold nanoparticles (Au NPs). The surface topography of the modified electrode was investigated using surface analysis techniques such as X-ray photoelectron spectroscopy (XPS) and scanning electron microscopy (SEM) combined with energy dispersive X-ray analysis (EDX). The sensor efficiency was validated by different electrochemical techniques such as differential pulse voltammetry (DPV) and cyclic voltammetry (CV). The voltammetric response of nilutamide was observed at −0.6 V in DPV, using Britton-Robinson buffer (pH 6.2) as the supporting electrolyte. The linear concentration range for nilutamide was 4.0 × 10^–8^ mol L^–1^–7.0 × 10^–7^ mol L^–1^ and 1.2 × 10^–6^ mol L^–1^–15.9 × 10^–6^ mol L^–1^ (LOD = 2.2 × 10^–9^ mol L^–1^). The recovery values for the electrochemical method ranged from 90.6% to 87.4%. The developed sensor and method were successfully applied to the analysis of nilutamide in tablet formulations, exhibiting satisfactory accuracy and precision.

## Graphical abstract

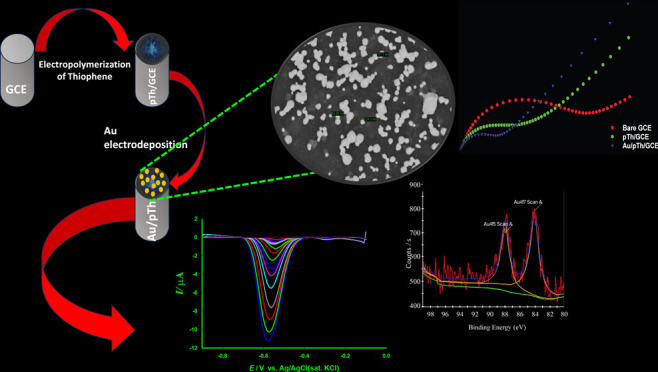

## 1 Introduction

Nilutamide (NLT) (5,5-dimethyl-3-(4-nitro3-(trifluoromethyl)­phenyl)­imidazolidine-2,4-dione) (Figure 1) is categorized as an antiandrogen medication and is commonly prescribed for the treatment of prostate cancer. NLT inhibits androgen receptor binding sites and control testicular function. However, overdosage of NLT can cause undesirable effects such as brain damage, neurological dysfunction, decreased musculature, decreased bone mass, flushing, interstitial pneumonia, computer-dependent fatigue, chest pain, and malfunction. Therefore, biomarker of NLT is quite important.^1–3^ Pharmacokinetic studies have reported that the maximum plasma concentration of NLT after therapeutic administration is approximately 1–3 μg mL^–1^. Therefore, the development of sensitive analytical methods capable of detecting nilutamide at these concentration levels is important for pharmaceutical analysis and therapeutic monitoring.^1^ In addition to the analysis of drug samples, the presence and analysis of pharmaceutical compounds in environmental media have become an important research topic in recent years. Pharmaceutical residues can be transported into aquatic environments and accumulate in environmental systems as a result of drug production processes, hospital waste, and the improper disposal of unused drugs. Although there is no specific maximum residue limit established for nilutamide in environmental media, monitoring pharmaceutical compounds in environmental samples is becoming increasingly important due to their potential ecotoxicological effects.^4,5^ Therefore, the need for an accurate and rapid method for the detection of NLT in different media. Several methods have been developed for the detection of NLT, including, chromatography,^2^ spectrophotometry,^3,6^ and electrochemical methods have been utilized for the determination of NLT.^7–16^ The electrochemical techniques are capable methods due to their wide sensitivity even at very lower analyte concentrations, short response time, low cost, ease of use, and selective detection of analytes.^8^ In electrochemical methods, polymers, metal nanoparticles, or carbon-based materials are used as modification materials. Gold nanoparticles stand out as an electrochemical sensor material due to their good electrical conductivity, large surface area, high sensitivity, and environmentally friendly nature.^9^ In addition to metal nanoparticles, polymers also have the ability to increase the sensitivity and surface area of the electrode.^10^ Furthermore, the selection of appropriate electrode material determines the efficiency of the electrode. For this reason, designing electrodes based on hybrid materials or sensors that enable the selective and sensitive detection of NLT is crucial for advancing voltammetric methods for its determination.

**Figure 1:**
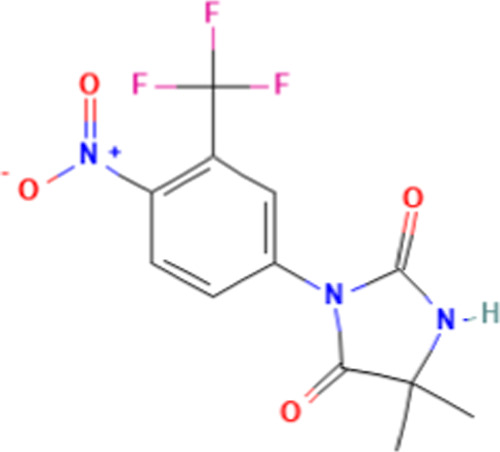
Chemical structure of nilutamide.

When examining studies on the voltammetric determination of NLT, it has been aimed to differentiate the electrode materials to achieve more sensitive determination. One of the first studies on the voltammetric determination of NLT was conducted by Temerk and colleagues in 2015.^7^ In this study, NLT detection was performed in blood and urea samples using a ZnO nanoparticles modified carbon paste electrode in a medium containing cetyltrimethylammonium bromide (CTAB). Various parameters, such as chemical and instrumentation, affecting the observed electroanalytical response was examined and optimized for the determination of NLM. Electrochemical parameters such as the surface concentration, electron transfer coefficient, and standard rate constant of NLT were calculated on the modified electrode. The obtained detection and quantification limits were 3.21 × 10^–9^ mol L^–1^ and 1.07 × 10^–8^ mol L^–1^, respectively, using square wave adsorptive stripping voltammetry (SWAdSV). In another study aimed at determining NLT, a sulfur-decorated reduced graphene oxide (S-rGO) composite material modified with Cu_2_V_2_O_7_ was used as the electrode material.^17^ In this study, NLT was determined with a high sensitivity of 26.26 μA μmol L^–1^ cm^–2^ and a low detection limit of 0.00459 nmol L^–1^ for a linear range of 0.001–15 μmol L^–1^. The electrode performance shown exceptional efficacy in the real-time monitoring of biological samples, including human blood serum and urine. In another study using graphene materials, WS_2_ nanostructures were synthesized on N,B-rGO, and this composite material was used for the amperometric determination of NLT.^18^ This composite electrode provided a wide linear range between 0.01 μmol L^–1^ and 250 μmol L^–1^, while demonstrating a detection limit of 0.003 μmol L^–1^. In recent years, metal–organic frameworks (MOFs), which are frequently used as electrode materials, have also been employed in NLT determination.^19^ In this study, Co–Ni–Cu-MOF was synthesized on nickel foam and allowed NLT determination with linear ranges of 0.5–70 μmol L^–1^ and 70–900 μmol L^–1^, and a detection limit of 0.48 n mol L^–1^.

The direct reduction of NLT on bare electrodes is very difficult due to positive peak potential and weak electron transfer processes. Therefore, as observed from previous studies, the electrode surface is quite important for sensitivity and selectivity in methods used for NLT determination. Nevertheless, because studies examining the electrochemical behavior of NLT and methods for its determination have been quite limited in the past 7 years, developing a selective and sensitive electroanalytical method is very important. Moreover, to perform more precise measurements, the electrode surface needs to be modified with a certain catalyst. Finding the right electrode material for an electrochemical sensor will be key to overcoming most of the challenges.

In this study, for the first time in the literature, the GCE surface was sequentially modified with polythiophene as a conductive polymer and gold nanoparticles via electrochemical techniques to improve the selectivity and sensitivity toward NLT detection. The developed Au/pTh/GCE-based sensor demonstrates considerable potential for the reliable determination of NLT in pharmaceutical sample matrices.

## 2 Experimental Section

### 2.1 Materials and Instruments

Sodium hydroxide (NaOH), nilutamide, thiophene (Th), ethanol (CH_2_CH_3_OH), hydrochloric acid (HCl), alumina (Al_2_O_3_), boric acid (H_3_BO_3_), phosphoric acid (H_3_PO_4_), acetic acid (CH_3_COOH) was purchased from Sigma-Aldrich and Merck. NLT solution was prepared daily with ultrapure water. Britton-Robinson buffer measured at different pHs was recorded as the supporting electrolyte.

A triple electrode system which is working electrode is glassy carbon electrode (GCE), Pt wire is auxiliary electrode and Ag/AgCl (sat. KCl) is comparison electrode that was used for voltammetric measurements, which are electrochemical impedance spectroscopy, cyclic voltammetry and differential pulse voltammetry methods in a suitable supporting electrolyte media in Autolab 101 Electrochemical Measurement devices under the nitrogen environment. Before each measurement, the solutions were purged with nitrogen gas to remove dissolved oxygen. WTW pH 330i brand pH meter was used for pH measurements. The surface of the GCE was polished with alumina and then cleaned up in a Bandelin Sonorex brand ultrasonic bath. Millipore Milli Q (18.2 MΩ) pure water device was used for ultrapure water. Scanning electron microscope (SEM) analyses were performed using a Zeiss Gemini 500 model device to examine the morphological characteristics of the modified electrodes. Crystal structure analyses were determined using a PANalytical Empyrean model X-ray diffraction (XRD) device. For the examination of surface elemental composition and chemical states, a Thermo Scientific K-Alpha model X-ray photoelectron spectroscopy (XPS) device was used. Additionally, electrochemical impedance spectroscopy (EIS) measurements were performed using the Autolab PGSTAT 302N potentiostat/galvanostat system (Metrohm Autolab, Utrecht, Netherlands). The High-performance liquid chromatography (HPLC) analyses were performed using an Agilent 1260 Infinity HPLC-DAD system.

### 2.2 Preparation of Au Nanoparticles Modified Polythiophene on Glassy Carbon Electrode

After the GCE surface was cleaned with a polishing material, the pTh was coated with 0.1 mol L^–1^ thiophene and 0.1 mol L^–1^ NaClO_4_ solution in acetonitrile by cyclic voltammetry. Cyclic voltammetric study tried various cycles versus Ag/AgCl in the 0.0 to 2.0 V potential range with 100 mV/s scan rate. The obtained voltammograms were showed in Figure S1A. The cyclic voltammetry curve presented in the figure clearly demonstrates that the monomer containing a thiophene unit polymerizes on the electrode surface via electrochemical oxidation. The three distinct peaks observed at 0.40, 0.70, and 1.40 V, respectively, during the anodic scan correspond to the stepwise oxidation processes of the monomer and the subsequent formation of the polymer film. The first anodic peak (at 0.40 V) represents the oxidation of the thiophene ring, the formation of a radical cation intermediate, and the subsequent initiation of oligomerization. The second anodic peak (at 0.70 V) can be associated with the further oxidation of the formed oligomeric species and the growth of conductive polymer chains on the electrode surface. The third anodic peak (at 1.40 V) is engaged with the future oxidation of the growing polymer film and/or the redox reactions of the oxidation products accumulated on the electrode surface.^20–22^ The polymerization mechanism of thiophene was showed in the Figure S1A. The increase in anodic peak currents up to the 12th cycle indicates that the polymer film has grown effectively on the electrode surface and has acquired an electrochemically active structure. The gradual decrease in current in the subsequent cycles (between the 12th and 30th cycles) can be explained by the restriction of mass transport due to the increase in film thickness, changes in the conductivity/doping level within the film, or partial passivation of the surface.

Au nanoparticles were deposited on pTh/GCE with different cycle numbers (between 3 to 20) by cyclic voltammetry in 1 mmol L^–1^ HAuCl_4_+ 0.1 mol L^–1^ HCl solution with 50 mV/s scan rate. The cyclic voltammograms obtained from Au deposition on pTh/GCE surface were illustrated in Figure S1B. In this voltammogram, the cathodic peak observed at −0.45 V in the cathodic scan can be linked to the reduction of Au^3+^ species. The steady increase in current throughout the cycles indicates that the gold layer has successfully adhered to the polythiophene surface, that the electroactive surface area has increased, and that the electron transfer kinetics of the electrode have improved. During scanning in the anodic direction, it shows that some of the Au species deposited on the surface as Au^0^ are reoxidized. Thus, stable metallic Au species have formed on the surface. The resulting electrode was named Au/pTh/GCE. The steps of electrode’s preparation were shown in Scheme 1.

**Scheme 1:**
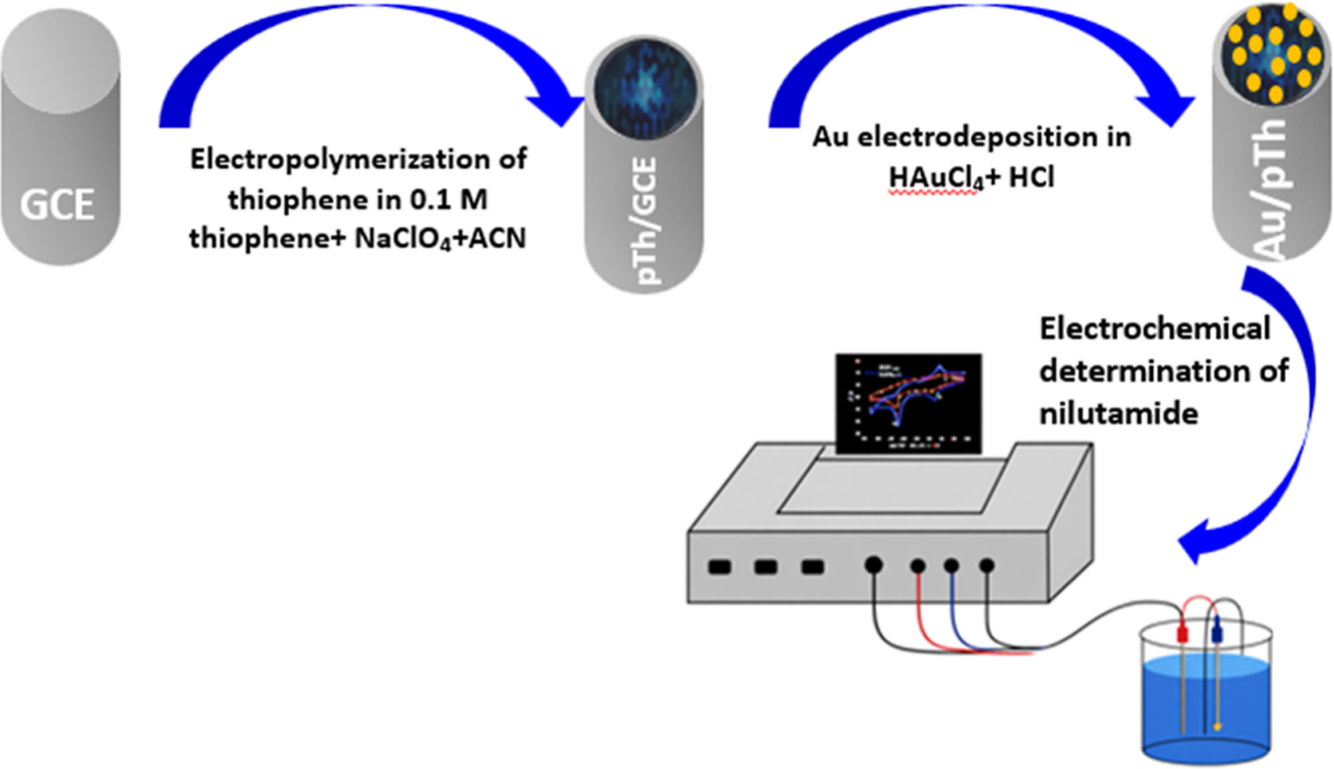
Schematic Illustration of the Fabrication of Au–PTh/GCE

**Scheme 2:**
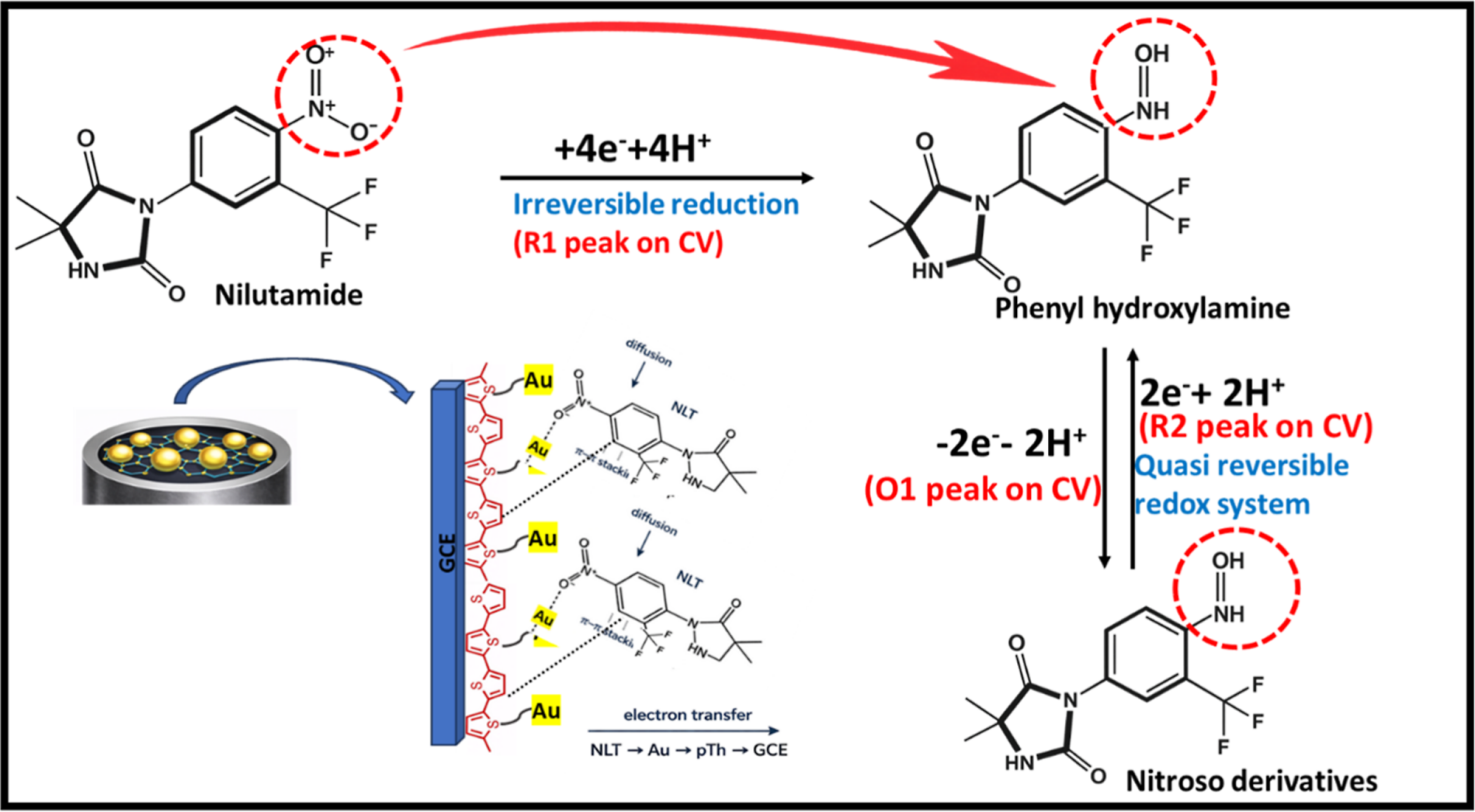
Electrochemical Redox Mechanism of NLT on Au/pTh/GCE and Interaction between Electrode Surface and NLT

## 3 Results and Discussion

### 3.1 Characterization of Modified Electrode

The morphologic and chemical characterization of pTh/GC and Au/pTh/GC electrodes were performed using SEM and XPS, respectively. Figure 2A indicates that the GCE surface was successfully modified with polythiophene (pTh), as evidenced by the homogeneous distribution of the polymer structures, which contributes to improved electrochemical performance. Figure 2B,C indicate the SEM images of gold (Au) nanoparticles modified on the pTh surface with different magnitude, where the Au nanoparticles are smoothly and densely distributed. The particle size of the Au nanoparticles was found to be nearly 80–130 nm. The polythiophene–Au nanocomposite provides high catalytic activity and enhanced conductivity by increasing the effective electrode surface area through the presence of these small, well-dispersed particles. Observed from the EDX spectrum shown in Figure 2D, the weight percentage (%*W*_t_) of carbon (C) is 21.72% and 78.28% gold (Au) nanoparticles both accumulated on GCE surface. In addition to this, the atom percentage of 81.98% carbon and of 18.02% gold nanoparticles means gold nanoparticles dominate the electrode surface.

**Figure 2:**
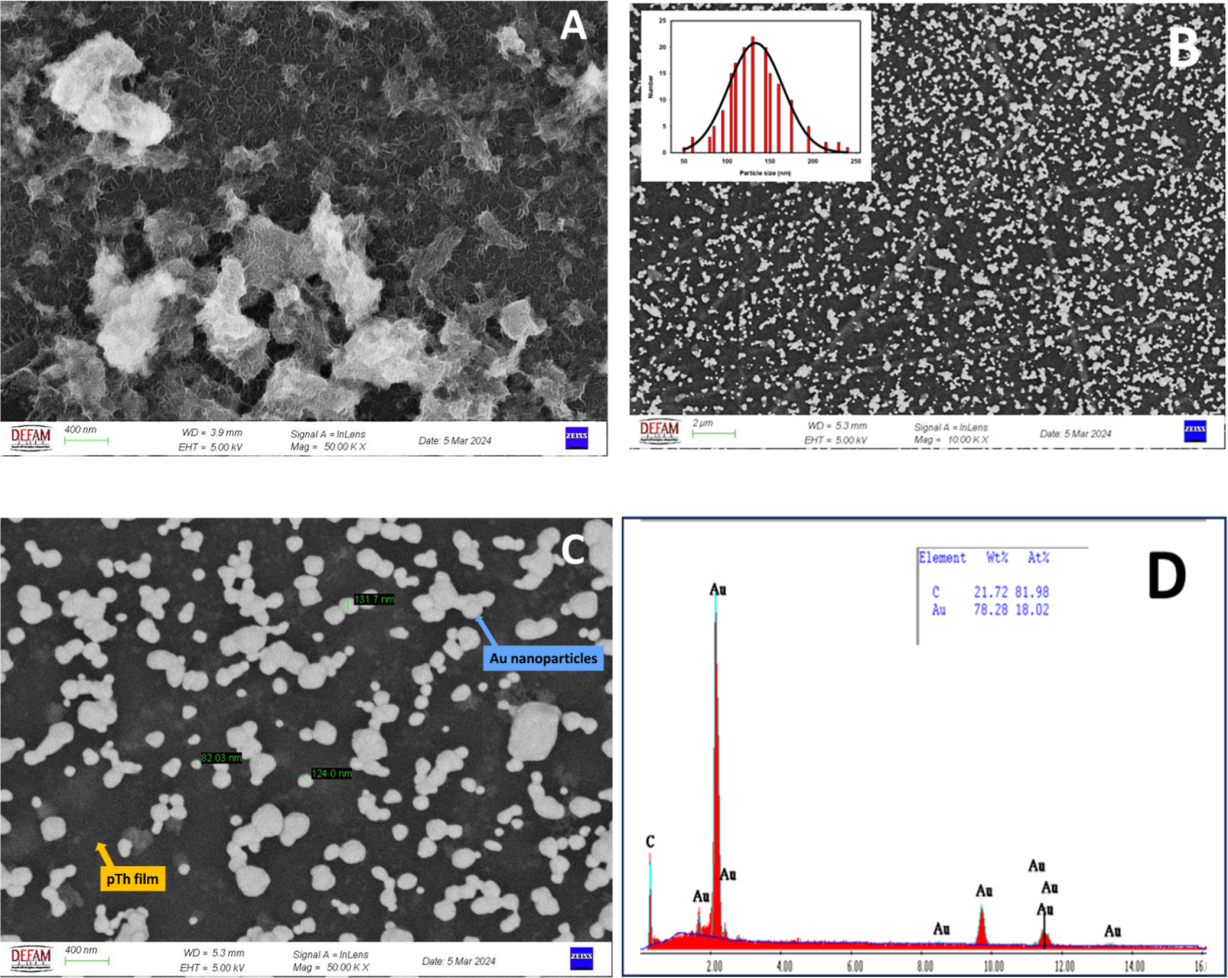
SEM images of (A) pTh/GCE (B) Au/pTh/GCE (×10000 magnitude), inset: the particle size distribution histogram of samples. (C) Au/pTh/GCE (×50000 magnitude), and (D) EDS spectrum of Au/pTh/GCE.

X-ray photoelectron spectroscopy (XPS) was used to elucidate the chemical structure of the material obtained by modification of the pTh surface with Au (Figure 3). The XPS survey spectrum in Figure 3A clearly reveals that the main elements present in the structure are on the GCE composite electrode modified with polythiophene and coated with Au nanoparticles. The prominent C 1s peak (∼285 eV) observed in the spectrum represents the carbon content of GCE and polythiophene layer. The O 1s peak (∼532 eV) confirms the presence of oxygen-containing functional groups on the surface. The S 2p peak (∼162–164 eV) shows the presence of sulfur atoms in the polythiophene structure and proves that the polymer was successfully coated on the electrode surface. The asset of gold nanoparticles was confirmed by the Au 4f peak (∼84–88 eV). These results, which show that gold metal nanoparticles were successfully deposited on the surface, reveal that GCE modified with polythiophene and Au nanoparticles were successfully prepared and the elements on the surface were effectively located. The S 2p XPS spectrum of the modified gold nanoparticle glassy carbon electrode on polythiophene shows the binding energies and chemical environment of the sulfur element (Figure 3B). The S 2p3/2 Scan A peaks seen in the spectrum are located at 163.29 eV, which represents the sulfur atoms bonded to the thiophene ring in the polythiophene structure. This binding energy shows that the sulfur atoms in the polymer have a stable structure with covalent bonds. The S 2p3/2 Scan B peaks are located at 167.82 eV and indicate a higher oxidation state of sulfur atoms. This peak indicates that sulfur is in different chemical environments on the surface and probably contains oxidized species. These different peaks in the spectrum confirm that sulfur is present in different binding modes in the polythiophene structure and that the polymer and gold nanoparticles are successfully placed on the surface. The Au 4f XPS spectrum of Au/pTh modified GCE shows the binding states of gold nanoparticles on the surface (Figure 3C). The Au 4f_7/2_ peak seen in the spectrum is located at 84.06 eV and the Au 4f_5/2_ peak is located at 88.14 eV. These binding energies show that the gold is in metallic form and has been successfully placed on the surface. The peaks of gold nanoparticles at these energy levels indicate that Au nanoparticles interact strongly with the polymer structure and form a stable bond with the chemical structure on the surface. These results confirm that the Au nanoparticles are effectively modified on the glassy carbon electrode surface and a suitable structure is provided for electrochemical applications. The C 1s XPS spectrum shows various bonding environments and chemical states of carbon on the surface (Figure 3D). The C 1s peak at 284.59 eV represents sp^2^ hybridized C=C bonds, indicating carbon–carbon bonds in graphitic carbon or thiophene ring. The C 1s peak at 286.14 eV indicates carbon atoms bonded to heteroatoms such as C–O or C–S, representing carbons interacting with sulfur atoms in the polythiophene structure. The C 1s peak at 287.63 eV indicates the presence of C=O groups, while the C 1s peak at 288.84 eV indicates bonding states associated with ester or carbonyl species.^23^

**Figure 3:**
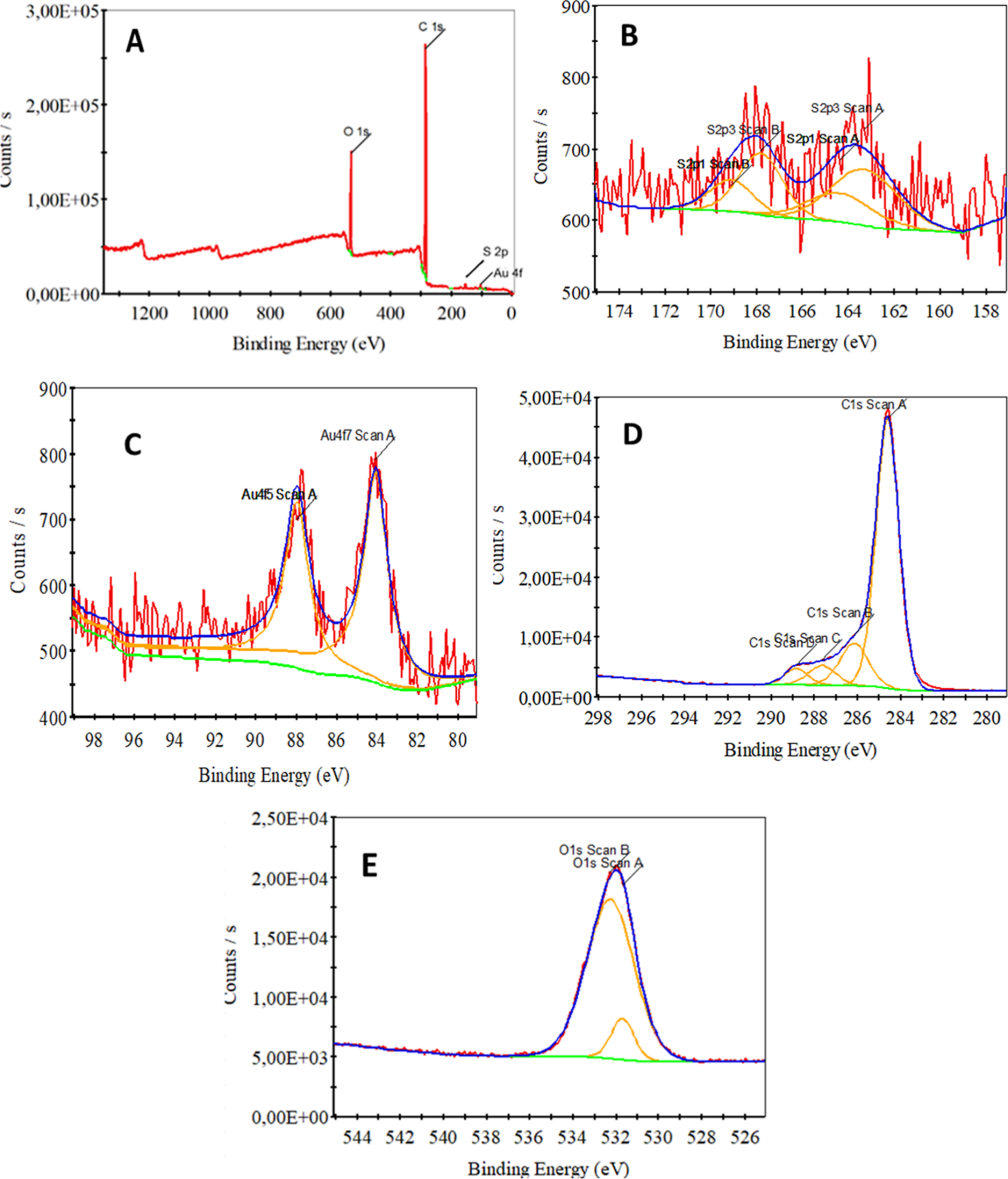
XPS spectrum of (A) Au/pTh/GC electrode survey; and overall spectrum of (B) S 2p, (C) Au 4f, (D) C 1s and (E) O 1s.

This spectrum confirms the existence of various carbon species on the surface and the successful placement of polythiophene and gold nanoparticles on the surface. The O 1s high-resolution XPS spectra presented in Figure 3E show oxygenated functional groups on the Au/pTh/GCE surfaces. Although oxygen is absent in the theoretical structure of pure polythiophene, the obtained O 1s signal has been deconvoluted into two main components. The first peak, centered at 531.68 eV, is attributed to carbonyl (C=O) groups and Sulphone/sulfoxide (−S­(=O)_2_–/ −S=O−) species formed as a result of the partial oxidation of thiophene rings during polymerization or modification processes. The second component at 532.25 eV represents hydroxyl (−OH) and ether (C–O–C) bonds on the surface, as well as water molecules physically adsorbed on the porous polymer matrix. These findings demonstrate the surface functionalization of the PTh matrix and the stable oxygen species formed through environmental interactions. The surface elemental composition of the Au/polythiophene electrode was determined from the XPS survey spectrum. The atomic percentages of the detected elements were found to be Au (16.37%), S (0.04%), C (77.32%), and O (10.32%). The high carbon content originates from the polythiophene polymer backbone, while the presence of oxygen species may be attributed to oxygen-containing functional groups and possible partial oxidation of the polymer during the electrochemical processes. The Au signal confirms the successful deposition of Au nanoparticles on the polythiophene-modified electrode surface. In conclusion, XPS analysis shows that polythiophene modified gold nanoparticle glassy carbon electrode was successfully prepared and carbon, oxygen, sulfur and gold elements were effectively located on the surface. This modification confirms that the surface structure of the electrode offers a suitable structure for electrochemical applications.

The investigate of the electrochemical behavior of the bare GCE, pTh/GCE, and Au/pTh/GCE was carried out using cyclic voltammetry (CV) at a scan rate of 0.05 V s^–1^ in 0.1 mol L^–1^ KCl solution with 5.0 mmol L^–1^ K_3_[Fe­(CN)_6_]/K_4_[Fe­(CN)_6_]. In Figure 4A, the GCE, pTh/GCE, and Au/pTh/GCE electrodes showed distinct oxidation and reduction peaks for the Fe^2+^/Fe^3+^ redox pair. Peak potential differences (Δ*E*p = *E*_pa_ – *E*_pc_) show the electrode’s ability to transfer electrons, for unmodified GCE, pTh/GCE and Au/pTh/GCE, these were 75 mV, 85 mV and 93 mV, respectively. After the electrode surface was modified with polythiophene, there was an increase in peak current and further current response enhancement was noted after the addition of Au nanoparticles to the polymer. Original electroactive surface area of electrodes was calculated using the Randles–Sevcik equation with a redox probe and was found to be 0.066 cm^2^, 0.086 cm^2^, and 0.113 cm^2^ for bare GCE, pTh/GCE, and Au/pTh/GCE respectively. The findings demonstrate that the alteration of the electrode surface has resulted in a significant increase in the electroactive surface area. The composite structure formed by the combination of Au nanoparticles with the polythiophene framework facilitates an expansion of the electrode’s active surface area, thus improving the electrochemical response. Electrochemical impedance spectroscopy (EIS) measurements were performed to investigate the interface properties of the electrodes. In the Nyquist diagrams given in Figure 4B, the semicircular part in the high-frequency region represents the charge transfer resistance (*R*_ct_), while the linear part in the low-frequency region shows diffusion-controlled processes. Impedance data were evaluated using the Randle equivalent circuit model. According to the results obtained, the bare GCE electrode had the highest charge transfer resistance at 475 Ω, while this value decreased to 225 Ω after modification with polythiophene. In the Au/pTh/GCE electrode obtained by adding Au nanoparticles to the polymer surface, the *R*_ct_ value was determined as 132 Ω. This significant decrease in charge transfer resistance shows that the composite structure formed by the combination of Au nanoparticles and polythiophene significantly accelerates electron transfer on the electrode surface. When the obtained CV and EIS results are evaluated together, it can be said that the polythiophene electrode modified with Au nanoparticles exhibits improved electrochemical performance thanks to its high electroactive surface area and low charge transfer resistance, and constitutes a suitable platform for electrochemical sensor applications.

**Figure 4:**
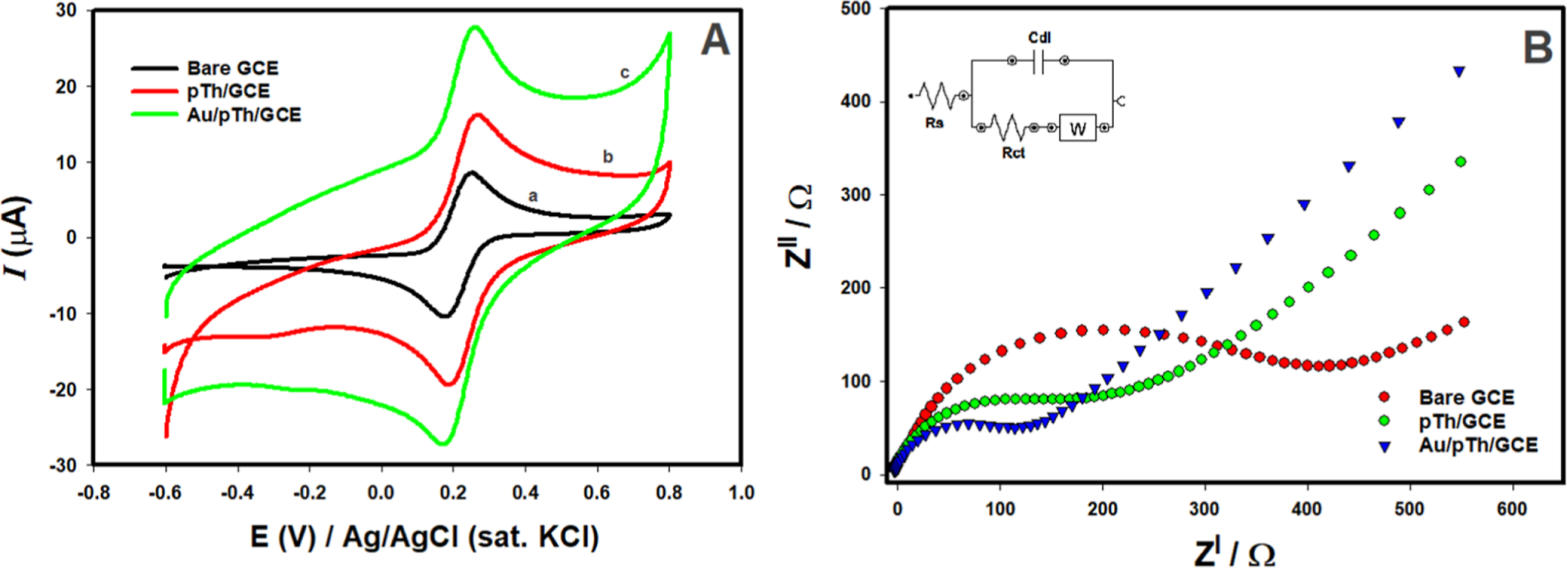
(A) CVs and (B) EIS of (a) bare GCE, (b) pTh/GCE, (c) Au/pTh/GCE in 0.1 mol L^–1^ KCl solution containing 5.0 mmol L^–1^ K_3_[Fe­(CN)_6_]/K_4_[Fe­(CN)_6_] with the frequencies swept from 0.02 to 100.0 × 10^3^ Hz at the formal potential. Inset is the Randles circuit model for the modified electrodes.

### 3.2 Electrochemical Behavior of Nilutamide

Cyclic voltammetric technique was used to study the electrochemical behavior of nilutamide (NLT) and the voltammograms of bare GCE, pTh/GCE, Au/GCE and Au/pTh/GCE in pH 6.2 Britton-Robinson buffer without (Figure 5A) and containing (Figure 5B) NLT were recorded at a scan rate of 0.05 V s^–1^. In the selected potential range without NLT solution, any oxidation or reduction peak was not observed for bare GCE, Au/GCE and Au/pTh/GCE, while a peak was observed at −0.28 V in pTh/GCE which is due to the polythiophene on the surface. At the same time, differences in the current heights of the supporting electrode were observed with the surface modification. In the presence of 3.0 × 10^–4^ mol L^–1^ NLT, a distinct and very sharp cathodic peak (R1) was observed at −0.49 V in the same potential range (Figure 5B) which is related to the direct reduction of nilutamide to phenyl hydroxylamine.^24^ In the anodic scan, no other oxidation peak corresponding to the R1 peak was observed, indicating that the process was irreversible. In addition, two more peaks were observed in pTh/GCE, Au/GCE and Au/pTh/GCE, which were named as R2 and O1. The O1/R2 redox couple at 0.05 V is due to the reversible behavior of phenyl hydroxylamine to nitroso derivatives.^25^ Since the increase in R1 peak was higher, all current and potential evaluations were made according to the R1 reduction peak. The cathodic peak current of NLT on Au/pTh/GCE was approximately 2.0, 1.5, and 4.0 times higher and much sharper than that on pTh/GCE, Au/GCE and bare GCE, respectively (Figure S2B). In addition, the cathodic potential of nilutamide on Au/pTh/GCE (−0.49 V) was lower than that on other modified electrodes such as pTh/GCE (−0.523 V) and plain GCE (−0.61 V). The overall results show that Au/pTh/GCE provided improved current response and lower reduction potential for nilutamide determination. Au/pTh modified GCE is a more innovative and attractive electrode material than other modified and bare electrodes for the detection of anticancer drug NLT.

**Figure 5:**
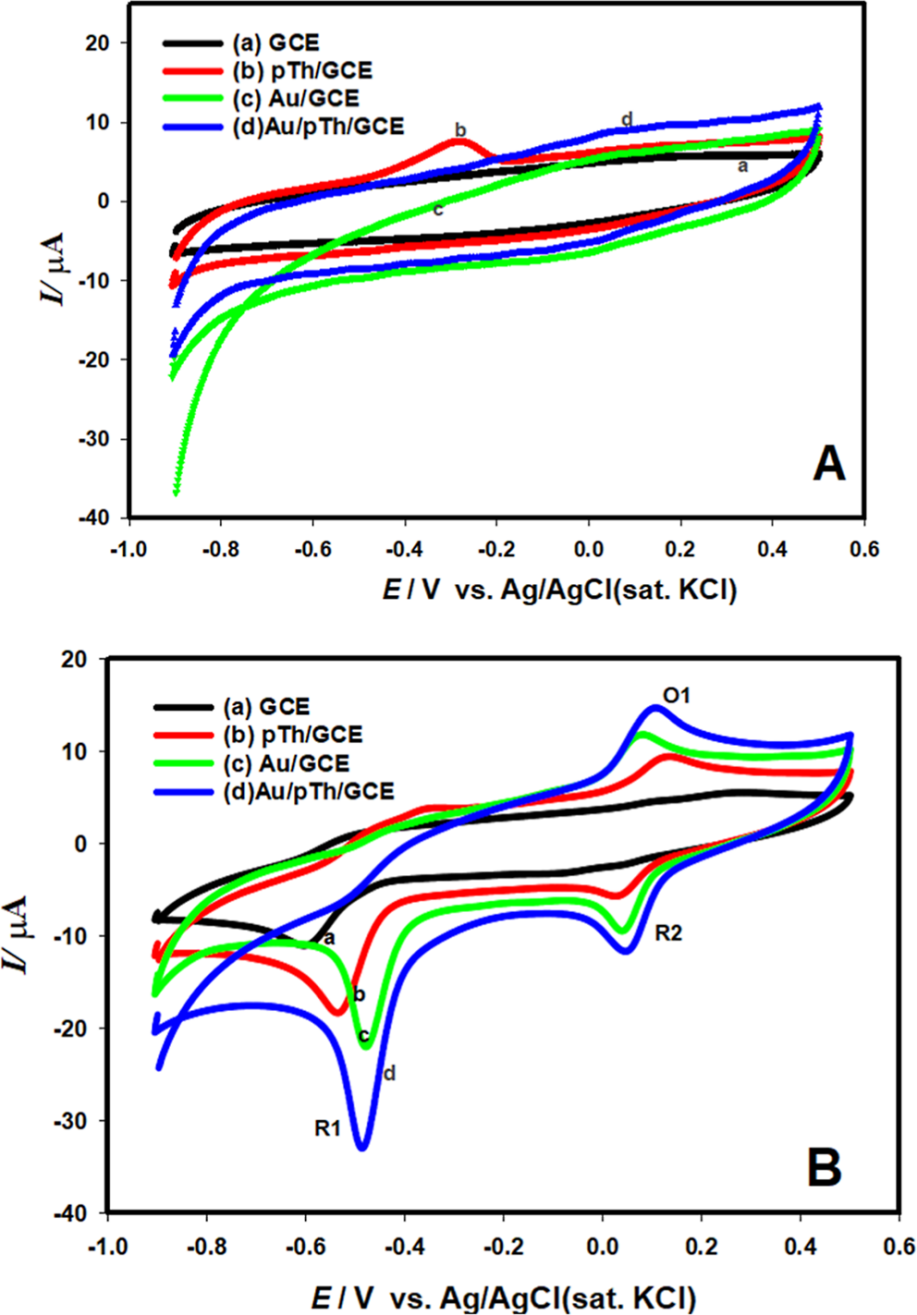
Cyclic voltammograms obtained in pH 6.2 BR buffer for different electrodes in the (A) absence and (B) presence (0.05 V s^–1^) of 3.0 × 10^–4^ mol L^–1^ NLT on (a) bare GCE (black line), (b) pTh/GCE (red line), (c) Au/GCE (green line) and (d) Au/pTh/GCE (blue line).

### 3.3 Optimization of Experimental Conditions

In order to obtain the maximum signal for NLT reduction, the number of cycles during polythiophene preparation and the number of cycles during the Au nanoparticle formation step were optimized using cyclic voltammetry. The effects of these parameters on the peak current of 3.0 × 10^–4^ mol L^–1^ NLT in pH 6.2 BR buffer are shown in Figure 6A and B. The highest peak current for NTL reduction was obtained at Au/pTh/GCE which prepared with 20 cycles for polythiophene, and 10 cycles for Au.

**Figure 6:**
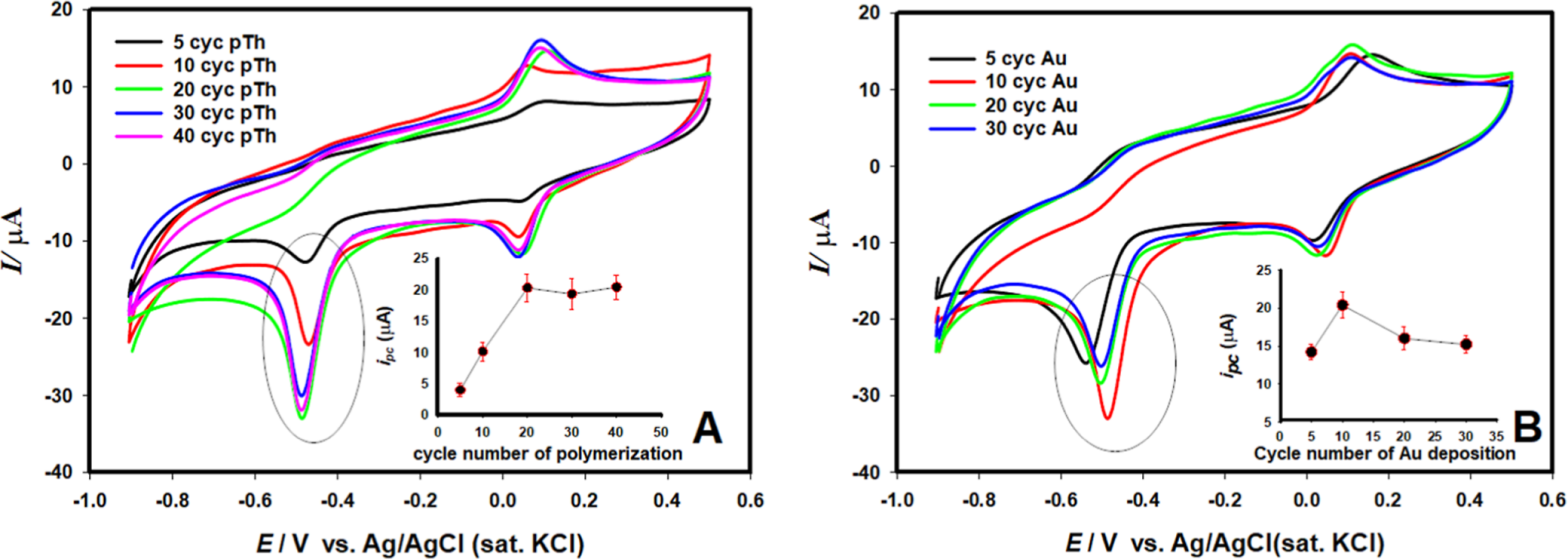
Optimization studies (A) cycle number of electropolymerization, (B) cycle number of Au nanoparticle deposition on PTh/GCE.

### 3.4 Effect of Supporting Electrolyte pH on Cyclic Voltammetric Behavior of Nilutamide

The effect of supporting electrolyte pH on the electrochemical peak current and potential of NLT was investigated using cyclic voltammetry in Britoon-Robinson buffers (pH 2.2–10.4) with different pH values containing 3.0 × 10^–4^ mol L^–1^ NLT on Au/pTh/GCE electrode (Figure 7A). Shifts in both peak current and potential of reduction peaks are observed with increasing pH. While the highest current density was obtained at pH 6.2, this peak current decreased significantly at pH 10.4, indicating that the electrochemical activity of NLT decreased with alkalinization of the medium. Therefore, pH 6.2 was selected as the optimum pH for further electrochemical studies. At higher pH values, hydroxyl ions interact more with nilutamide, causing deprotonation, which leads to a decrease in electrocatalytic activity. The linear plot of cathodic peak potential with different pH values is illustrated in Figure 7B. As the pH value increased, the cathodic peak potential shifted to the negative side and showed a linear trend. The linear equation is given by the cathodic peak potential (*E*_pc_ = −0.0593 pH + 1.4918, *R* ^2^ = 0.9956), which indicates that an equal number of protons and electrons are transferred in the reduction of nilutamide to hydroxylamine. Figure 6B shows that the reduction potential (*E*_pc_) shifts linearly to more negative values with increasing pH (*E*_pc_ = −0.0573 pH −0.207, *R* ^2^ = 0.9932), indicating that the electrochemical reduction of NLT is associated with proton transfer. Since the reduction of NLT is a proton-participating reaction involving the reduction of the nitro group to an aryl hydroxylamine derivative, the pH of the medium directly affects the electrochemical behavior. As the pH increases, the proton concentration in the medium decreases, and more negative potentials are required for the reduction reaction to occur. Therefore, it has been observed that the cathodic peak potential shifts to more negative values with increasing pH. The slope of this line is 0.0573 V/pH, indicating that an equal number of protons and electrons are present in the electrode reaction. At the same time, the peak value (*I*_pc_) reached its highest level at pH 6.2 and started to decrease at higher pH values. These results show that pH plays a crucial role in the electrochemical activity of NLT in terms of both potential and current. Considering all these results, pH 6.2 was selected as the supporting electrolyte in further studies.

**Figure 7:**
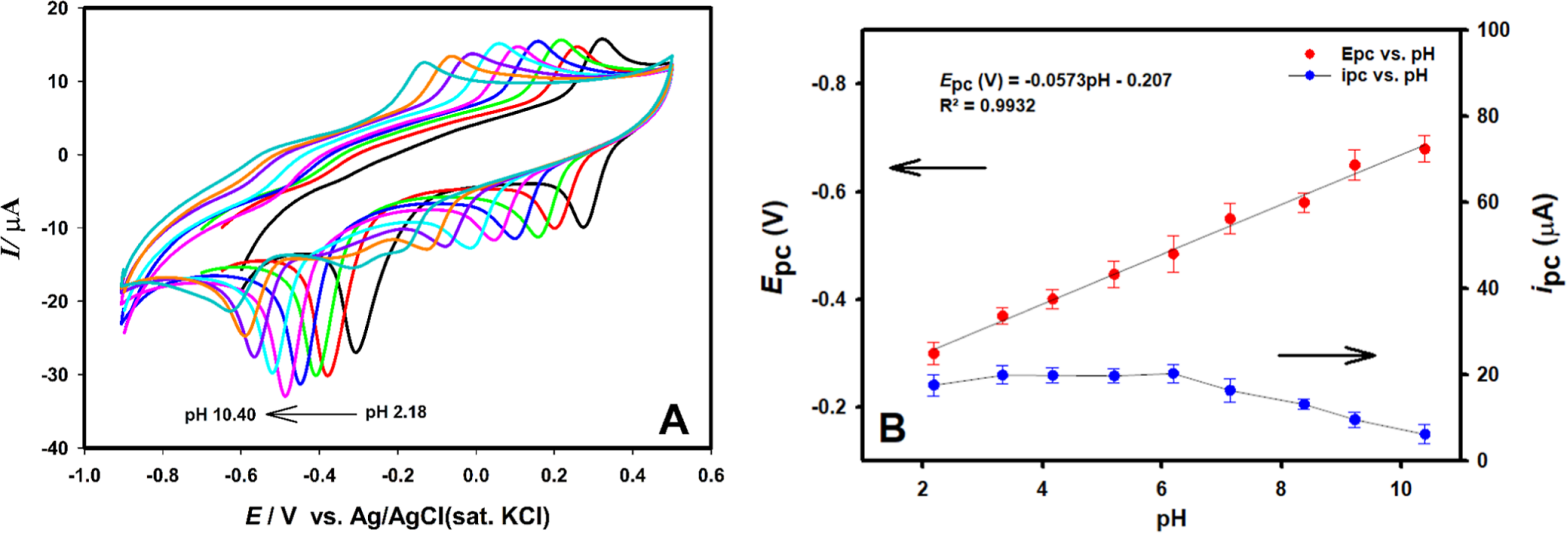
(A) Cyclic voltammograms of 3.0 × 10^–4^ mol L^–1^ NLT in Au/pTh/GCE in supporting electrolytes of different pHs (pH 2.2–10.4), (B) effects of pH value on the peak current and peak potential response of NLT (scan rate: 50 mV s^–1^).

### 3.5 Effect of Scan Rate on the Electrochemical Behavior of Nilutamide

To get information about the reaction kinetics of NLT reduction on the Au/pTh/GC electrode surface, cyclic voltammogram was acquired in the presence of 3.0 × 10^–4^ mol L^–1^ NLT in pH 6.2 BR solution under different scan rates (10.0–350.0 mV s^–1^) (Figure 8A). From the voltammograms, a linear relationship was obtained between the reduction peak currents (*I*_pa_) of NLT and the square root of the scan rate (*v* ^1/2^), and it was expressed as (*I*_pc_ = 2.8554 *v* ^1/2^ + 4.2372, *R* ^2^ = 0.9983) (Figure 8B). The linearity of this curve provides an easy understanding of the diffusion-controlled reduction of NLT on the Au/pTh/GCE electrode. In addition, the *E*_pc_ value of NLT on Au/pTh/GCE shifted to more positive potential as the scan rate increased. A linear correlation was formed between the reduction potentials (*E*_pc_) and ln *v* (natural logarithm of the scan rate) and was expressed as (*E*_pc_ = −0.0222 ln *v*-0.476, *R* ^2^ = 0.9990) (Figure 8C). This linearity shows that the reduction reaction that occurs at −0.49 V of NLT is irreversible. *E*_pc_ is described by Laviron’s equation^26^ for an irreversible reaction (eq 1)­

1 $$E_{p}=E^{0^{\prime }}+\frac{RT}{anF}\ln v$$

**Figure 8:**
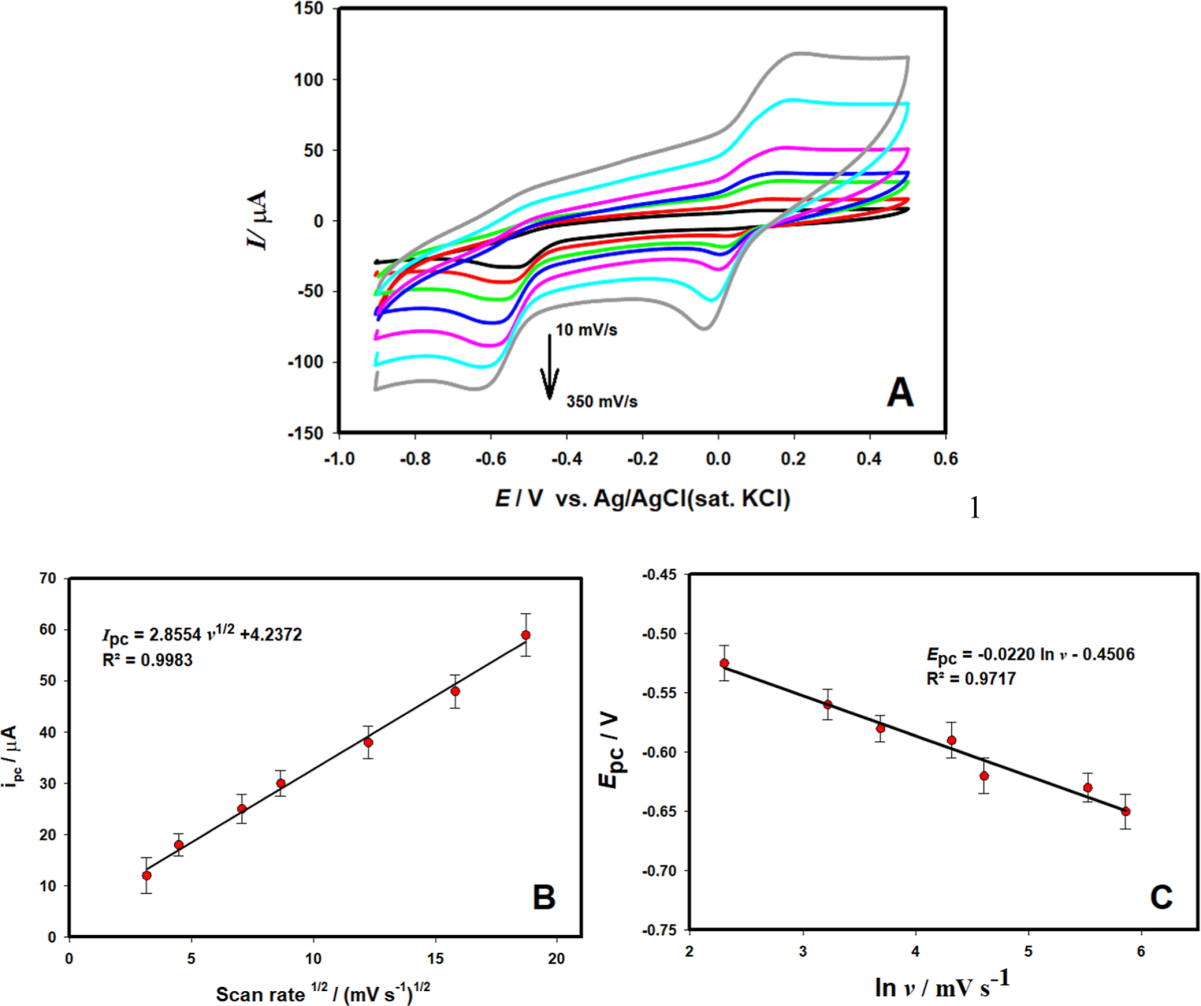
(A) CVs at different scan rates (10–350 mV s^–1^) in pH 6.2 BR solution containing 3.0 × 10^–4^ mol L^–1^ NLT on Au/pTh/GCE (B) *I*_pc_-*v* ^1/2^ curve, (C) ln *v*-*E*_pc_ curve.

According to the Laviron’s equation, the peak potential (*E*_p_) in irreversible electrode reactions exhibits a logarithmic relationship with the scan rate. In this equation, *E*_0_′ represents the formal potential, *R* represents the universal gas constant (8.314 J K^–1^ mol^–1^), *T* represents the absolute temperature (K), *F* represents Faraday’s constant (96.485 C mol^–1^), α represents the charge transfer coefficient, and n represents the number of electrons participating in the reaction. The slope of the linear relationship between *E*_p_ and ln ν corresponds to the expression RT/αnF. The α value was calculated using the slope of the experimentally obtained *E*_p_–ln ν graph. Furthermore, according to Bard and Faulkner, at 25 °C, a 10-fold increase in scan rate results in a shift of approximately 30/α mV in the peak potential (1.15RT/αF). Using this relationship, the charge transfer coefficient was found to be 0.31. The transferred electron number (*n*) in the NLT irreversible reduction reaction was found as 3.7, which can be reasonably approximated to 4 electrons.

This analysis designates that the first cathodic peak (R1) corresponds to an irreversible reduction process involving approximately four electrons. However, the electrochemical behavior of NLT is not limited to this irreversible step. As seen in the Figure 5B, an additional anodic/cathodic peak pair (O1/R2) was observed, suggesting the presence of a quasi-reversible redox process following the initial reduction step. Therefore, to gain further insight into the overall reaction mechanism, the O1/R2 redox couple was also evaluated using the dependence of peak potential on pH of supporting electrolyte and scan rate. For the quasi-reversible O1/R2 system, the linear relationships between peak potential and ln ν were expressed as *E*_p_(O1) = 0.0205 ln ν + 0.0541­(*R* ^2^ = 0.9961)­and *E*_p_(R2) = −0.02060 ln ν + 0.01017­(*R* ^2^ = 0.9917) (Figure S3A). According to Laviron’s equation for quasi-reversible systems, the slopes of the anodic and cathodic peaks correspond to *RT*/α*nF*and *RT*/(1-α)*nF*, respectively. From these values, the charge transfer coefficient (α) was calculated to be approximately 0.52, while the number of electrons involved in the O1/R2 redox couple was found to be about 2.4. Moreover, the heterogeneous electron transfer rate constant (*k* ^0^) was calculated using the Laviron equation based on the intercept and slope of the *E*_pa_ vs ln *v* plot, resulting in a value of approximately 1.75 × 10^–1^ s^–1^. The obtained value indicates that the electron transfer process occurring on the Au/pTh/GCE electrode surface is moderately fast. This suggests that electron transfer is facilitated by the conductive structure of the polymer matrix and the Au nanoparticles. In addition, the pH-dependent peak potential shifts for O1 and R2 were close to the theoretical Nernstian value of 59 mV pH^–1^ (Figure S3B), indicating that the number of protons involved is equal to the number of electrons transferred in this step. Accordingly, the quasi-reversible redox couple can be assigned to a 2*e* ^–^/2*H* ^+^transformation between the aryl hydroxylamine and nitroso intermediate. When the four-electron irreversible reduction step (R1) and the subsequent two-electron quasi-reversible redox transformation (O1/R2) are considered together, the overall electrochemical mechanism of NLT is consistent with a total six-electron, six-proton process. The measured values show good consistency with literature data regarding the electrochemical reaction of nilutamide.^14,28^ The possible redox reaction of NLT was shown in Scheme 2. In addition to the proposed redox mechanism, it is also important to consider the interactions occurring at the electrode interface. These interfacial interactions play a key role in governing the electrochemical response of NLT. The enhanced electrocatalytic performance of the Au/pTh/GCE electrode stems from the synergistic interaction between polythiophene (pTh) and Au nanoparticles. The π-conjugated structure of pTh supports efficient electron transport across the electrode surface while also facilitating the molecule’s proximity to the electrode surface through π–π interactions with the aromatic structure of NLT. On the other hand, Au nanoparticles accelerate electron transfer kinetics and contribute to the electrochemical reaction occurring at lower overpotentials due to their high electrical conductivity and catalytically active surface properties. Additionally, the homogeneous distribution of Au nanoparticles within the polymer matrix increases the electrode’s effective surface area, enabling the formation of more active sites. The structure formed by these two components improves both mass transport and charge transfer processes, leading to a significant increase in the electrochemical response of the NLT.

Chronoamperometric measurements were performed to determine the diffusion coefficient associated with the reduction process of NLT. The measurements shown Figure S4 were conducted at a potential of −0.60 V, where the reduction reaction of NLT was complete. In the obtained chronoamperometric curves, a decrease in current over time was observed following the potential step. This behavior is consistent with the typical diffusion-controlled process resulting from the depletion of the electroactive species near the electrode surface and its transport via diffusion from the solution. The diffusion coefficient was calculated from the slope of the curve between t^–1/2^ and current using the Cottrell equation (eq 2).^27^

2 $$I\left(t\right)=\frac{nFAD^{1/2}C}{\pi^{1/2}t^{1/2}}$$

in this equation, *n* represents the number of electrons participating in the reaction, *F* represents the Faraday constant, *A* represents the electrode surface area, *D* represents the diffusion coefficient, *C* represents the analyte concentration, and *t* represents time. During the analysis, the initial short time interval was disregarded to minimize the effect of the double-layer charge current. A linear relationship between *t* ^–1/2^ and *I* was obtained for the selected time interval (Figure S3-inset). The diffusion coefficient of NLT was calculated using the slope of the linear graph and found to be 5.5 × 10^–5^ cm^2^ s^–1^. This value indicates that mass transport is effective on the Au/pTh/GCE electrode surface and that the modified electrode possesses a high effective surface area.

### 3.6 Calibration Curves of Au/pTh Modified GCE toward NLT Determination

DPV was used in pH 6.2 BR buffer solution to study the analytical performance for NLT determination of Au/pTh/GCE. The DPV parameters, including pulse amplitude, and scan rate, were optimized to obtain the highest peak current and best peak shape as 0.01 and 0.020 V s^–1^, respectively (Figure S5). In optimum conditions, DPVs were recorded in the concentration range from 4.0 × 10^–8^ mol L^–1^ to 15.9 × 10^–6^ mol L^–1^ for NLT reduction (Figure 9). The reduction peak currents (*i*_pc_) of NLT were found to be proportional to the concentration over two linear ranges: 4.0 × 10^–8^ mol L^–1^–7.0 × 10^–7^ mol L^–1^ and 1.2 × 10^–6^ mol L^–1^–15.9 × 10^–6^ mol L^–1^ (Figure 9 inset), respectively, from the linear regression equations *i*_pc_ (μA) = 0.5174 *C*_NLT_(μmol L^–1^) + 3.037 (*R* ^2^ = 0.9978) and *i*_pc_ (μA) = 2.9339 *C*_NLT_ (μmol L^–1^) + 0.1082 (*R* ^2^ = 0.9978) were obtained. The limit of determination (LOD) of NLT reduction on Au/pTh/GCE was calculated with the equation LOD = 3.3 σ/m (s: standard deviation of the response for the supporting electrolyte, m: slope of the calibration curve) and was found to be 2.2 × 10^–9^ mol L^–1^. The limit of quantification (LOQ) value was calculated according to the equation LOD = 10 σ/m and found to be 7.3 × 10^–9^ mol L^–1^, whereas the LOD was 2.2 × 10^–9^ mol L^–1^. The high sensitivity obtained shows the success of the electrode synthesized by electrochemical processes against the accurate determination of nilutamide. The analytical performance of the developed electrode is presented in Table 1 in comparison with nilutamide sensors reported in the literature. As can be seen from the table, the electrode developed in this study demonstrates performance comparable to that of sensors reported in the literature in terms of sensitivity. However, its simple and rapid synthesis process, minimal use of chemicals, and lack of complex preparation steps offer significant advantages. Furthermore, the high selectivity achieved even if in the presence of interfering species demonstrates the electrode’s suitability for practical and reliable applications. In these respects, the developed electrode represents a strong alternative to existing approaches in literature, not only in terms of analytical performance but also in terms of applicability.

**Figure 9:**
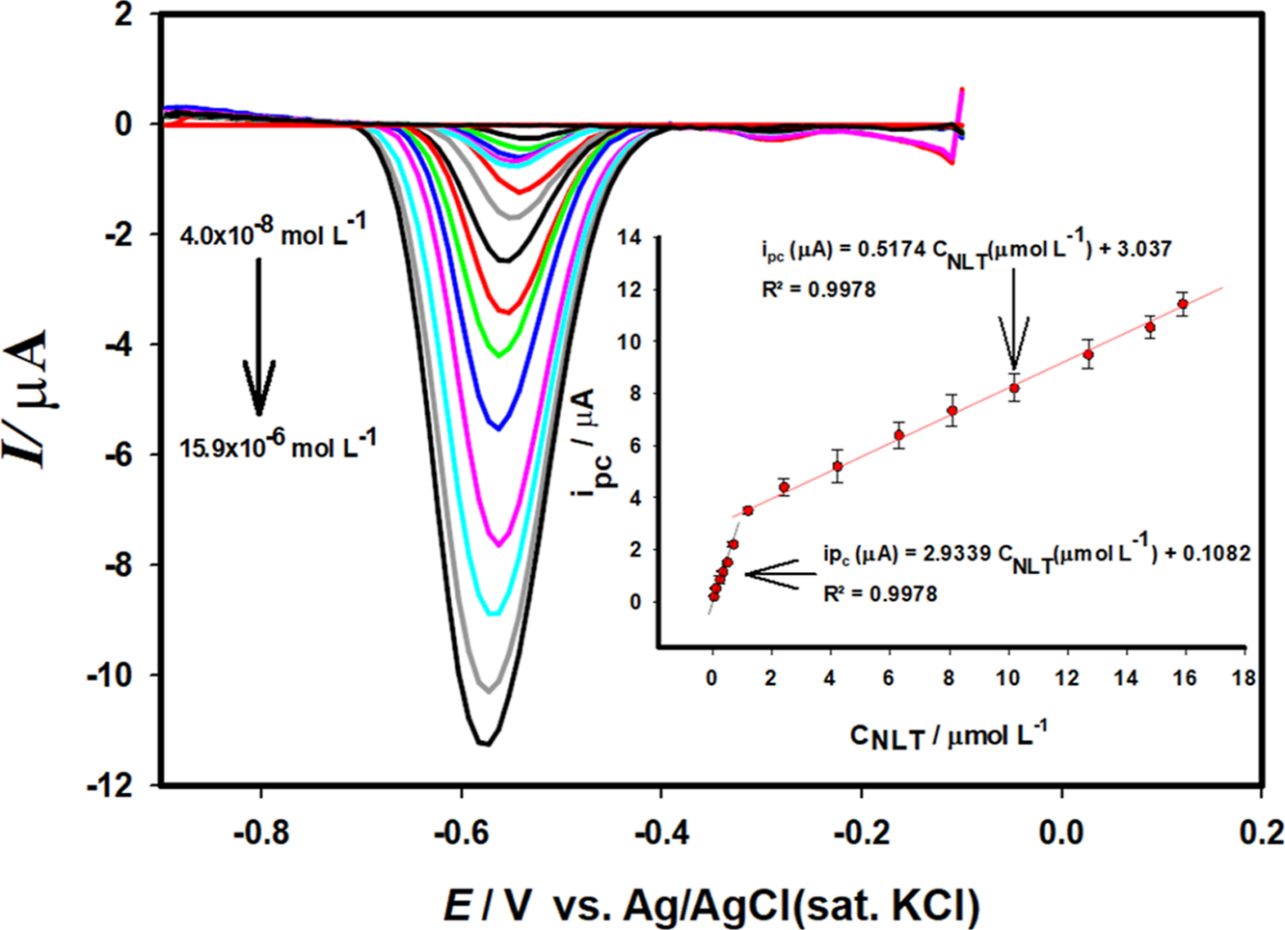
DPVs of NLT at different concentrations in Au/pTh/GCE. Inset: Calibration curve.

**Table 1:** Comparison of the Performance of the Proposed Electrode with Other Reported Electrochemical Nilutamide Sensors

electrode	method	LOD (nmol L^–1^)	sensitivity (μAμM^–1^ cm^–2)^	lineer range (μmol L^–1^)	ref
ZnONPs/CPE	SWAdSV	3.21	-	0.076–0.305	^7^
CeV/CNF	DPV	2.00	1.36	0.010–540	^13^
NiAl-LDH	DPV	5.00	15.6	0.0290–1543.8	^29^
CuCo2O4/MWCNT	DPV	10.0	1.50	0.01–170	^30^
SWNPs/SPCE	DPV	2.60	0.919	0.05–318	^28^
Au/pTh/GCE	DPV	2.20	4.58	0.04–0.70 and 1.20–15.9	This work

### 3.7 Interference Study, Reproducibility and Long-Term Stability Analysis

The selectivity of the Au–PTh/GCE was investigated using DPV in the presence of 2 μmol L^–1^ NLT and a 10-fold higher concentration of potential interfering species. In this context, biological compounds such as ascorbic acid (AA), dopamine (DP), uric acid (UA), glucose (Glu), catechol (CAT); some organic compounds containing nitro groups such as 4-aminophenol (4-AP), nitrobenzene (NB), 4-nitrophenol (4-NP) and metal ions such as Na^+^, K^+^, Ca^2+^, Mg^2+^ were added to the system separately. The fact that the change observed in the current responses obtained after the addition of interfering species remained below 10% (Figure S6A) indicates that the Au–PTh/GCE electrode exhibits high selectivity toward the target analyte and is highly resistant to interference effects. To evaluate the reproducibility of the Au–PTh/GCE, electrochemical responses for NLT reduction were recorded using three different composite electrodes produced using the same preparation procedures. In this context, intraday (*n* = 3) and interday (*n* = 3) measurements were performed, and relative standard deviation (RSD) values were calculated from the DPV data obtained in the presence of 2.0 μmol L^–1^ NLT in pH 6.2 BR buffer solution. The RSD values for intraday and inter-day measurements were found to be 3.77% and 5.64%, respectively (Figure S6B,C and Supporting Information). These results demonstrate that composite electrode has good reproducibility. The long-term stability of the Au–PTh/GCE electrode was evaluated by monitoring the time-dependent change in the reduction peak current for 2.0 μmol L^–1^ NLT. Throughout the long-term stability studies, the electrode was stored under standard conditions in a pH 6.2 BR buffer solution. At the end of the 12 days monitoring period, the decrease in the reduction peak current obtained for 2.0 μmol L^–1^ NLT was observed to be less than 10.9% (Figure S6D. These results demonstrate that the proposed electrode has sufficient stability for long-term voltammetric applications.

### 3.8 Determination of NLT in Drug Samples

The analysis of real samples plays a significant role in assessing the practical usefulness of a sensor. Since it is an NTL-sensitive electrode material even in the presence of biological components and metal ions, and it demonstrates good analytical performance with its ability to provide cheaper and faster analysis compared to expensive chromatographic methods. For this purpose, two nilutamide tablets with a label value of 50 mg (purity 88.5%) were ground into a homogeneous powder. An appropriate amount of the resulting powder was dissolved in a mixture of 15 mL ethanol and 25 mL distilled water in an ultrasonic bath. To remove insoluble excipients, the mixture was filtered, and the clear filtration was transferred to a volumetric flask and diluted appropriately. A specific volume was taken from the prepared sample solution and added to a pH 6.2 Britton–Robinson buffer solution (10 mL), and the analyses were performed using the square wave voltammetry method. The standard addition method was applied to minimize possible matrix effects, and the results obtained are presented in Table 2. The amount of nilutamide determined in tablets with a label value of 50 mg was found to be 43.2 mg. In order to further support the analytical performance of the proposed method, the determination of NLT in tablet samples was also carried out using a high-performance liquid chromatography (HPLC) method.^31^ The analysis was performed using a C18 reverse-phase column under isocratic conditions with a mobile phase consisting of water, methanol, and acetonitrile (40:50:10, v/v/v), a flow rate of 1.2 mL min^–1^, and an injection volume of 20 μL. For sample preparation, tablet powder corresponding to 10 mg of NLT was accurately weighed and diluted to 10 mL with methanol/water (45:55, v/v). The obtained solution was further diluted to 200 mL prior to analysis. Based on the calibration curve, the amount of NLT was calculated as 45.1 mg per tablet, corresponding to a recovery of 90.2% relative to the labeled content (50 mg per tablet) with a relative standard deviation (RSD) of 0.69%. In comparison, the electrochemical method showed recovery values in the range of 87.4–97.4% with RSD values between 2.9% and 5.2%. These results indicate that both methods provide acceptable accuracy and precision for the determination of NLT for routine pharmaceutical samples.

**Table 2:** NLT Tablet Analysis Using Au/pTh/GCE

sample	added (mg)	found (mg)	recovery (%)	RSD (%)
tablet	0	43.7	87.4	4.9
	10	57.3	95.5	3.7
	20	68.2	97.4	5.2
	30	72.5	90.6	2.9

## 4 Conclusions

In this study, a polythiophene (pTh) film was first formed on the GCE surface via electropolymerization for the electrochemical determination of nilutamide. Subsequently, gold nanoparticles were electrochemically deposited onto this layer to develop the Au/pTh/GCE electrode. SEM, EDS, and XPS analyses confirmed the homogeneous distribution of the polymer film and the successful deposition of metallic Au(0) species on the surface. The developed electrode showed a markedly enhanced current response along with a lower reduction potential for nilutamide. Under optimal conditions (pH 6.2 BR buffer), two linear working ranges were obtained in DPV analyses: 4.0 × 10^–8^–7.0 × 10^–7^ mol L^–1^ and 1.2 × 10^–6^–15.9 × 10^–6^ mol L^–1^. The limit of detection (LOD) was calculated as 2.2 × 10^–9^ mol L^–1^. The electrode showed good selectivity; signal changes in the presence of interfering substances remained below 10%. In the repeatability study, RSD values were found to be 3.77% intraday and 5.64% interday, and in the 12 day stability test, the current loss was below 10.9%. In tablet sample analyses, recovery values were obtained in the range of 87.4–97.4%. In conclusion, the electrochemically prepared Au/pTh/GCE electrode offers an effective, cheap and applicable sensor platform for the sensitive and selective determination of nilutamide, featuring a simple synthesis process, low detection limit, wide linear range, and satisfactory stability characteristics.

## Supplementary Material



## Notes

Cyclic voltammograms of thiophene electropolymerization and Au^3+^ deposition processes; comparison of peak current responses at different electrodes (bare GCE, pTh/GCE, Au/pTh/GCE) with error analysis; lnν–Epc and pH–Ep relationships for quasi-reversible redox peaks; chronoamperometric responses and corresponding Cottrell plots; optimization of DPV parameters including pulse amplitude and scan rate; interference studies with common electroactive species and ions; reproducibility (intraday and interday) and long-term stability data for NLT determination (PDF)
