# Supplementary material for: Electrochemical Determination of the Prostate Anti-Cancer Drug Nilutamide Using Gold Nanoparticle–Polymer Based Electrode
Source: ACS Omega. 2026 Apr 25;11(18):27449–61. doi: 10.1021/acsomega.6c01799 (PMC13177243; doi:10.1021/acsomega.6c01799)
Supplement: Supplementary file 1 [file ao6c01799_si_001.pdf]

Electrochemical Determination of the Prostate Anti-Cancer Drug Nilutamide Using Gold Nanoparticle–Polymer Based Electrode

B. Büşra KARAKAŞ, Z. Yaren Şahin, Aydan ELÇİ, Şükriye KARABİBEROĞLU*, Zekerya Dursun

Ege University, Faculty of Science, Department of Chemistry 35100 Bornova, İzmir Turkey

List of supplementary figures

Figure S1. A) CVs of 0.1 M thiophene in $NaClO_4$ + ACN on GCE; B) Cyclic voltammograms Au^{3+} ions in HCl solution on PTh/GCE. (Illustration created by the authors using ChemDraw and Microsoft Paint.)

Figure S2. Comparison of peak current responses obtained from different electrodes (bare GCE, pTh/GCE, and Au/pTh/GCE) in the presence of 3.0×10^{-4} mol L^{-1} NLT in pH 6.2 BR buffer. Error bars represent the standard deviation of replicate measurements.

Figure S3. A) lnv-E_{pc} and B) pH-Ep curves for O1/R2 quasi reversible peak in 3.0×10^{-4} mol L^{-1} NLT + pH 6.2 BR solution on Au/pTh/GCE.

Figure S4. Chronoamperometric curves for NLT obtained at a potential of −0.60 V, inset: the corresponding Cottrell plot ($t^{-1/2}$-I)

Figure S5. Optimization of differential pulse voltammetry (DPV) parameters for the electrochemical determination of NLT at the Au/pTh/GCE electrode in pH 6.2 BR buffer. (A) Effect of pulse amplitude and (B) effect of scan rate on the reduction peak current of NLT.

Figure S6. A) Interference studies of the developed Au-PTh/GC electrode for the 2.0 µmol L^{-1} NLT only or with 20.0 µmol L^{-1} AA, DP, UA, Glu, CAT, 4-AP, NB, NP, Na^+, K^+, Ca^{2+}, Mg^{2+} respectively; reproducibility studies B) intraday-measurements and C) inter-day measurement, D) long term stability studies for 2.0 µmol L^{-1} NLT in pH 6.2 BR buffer.

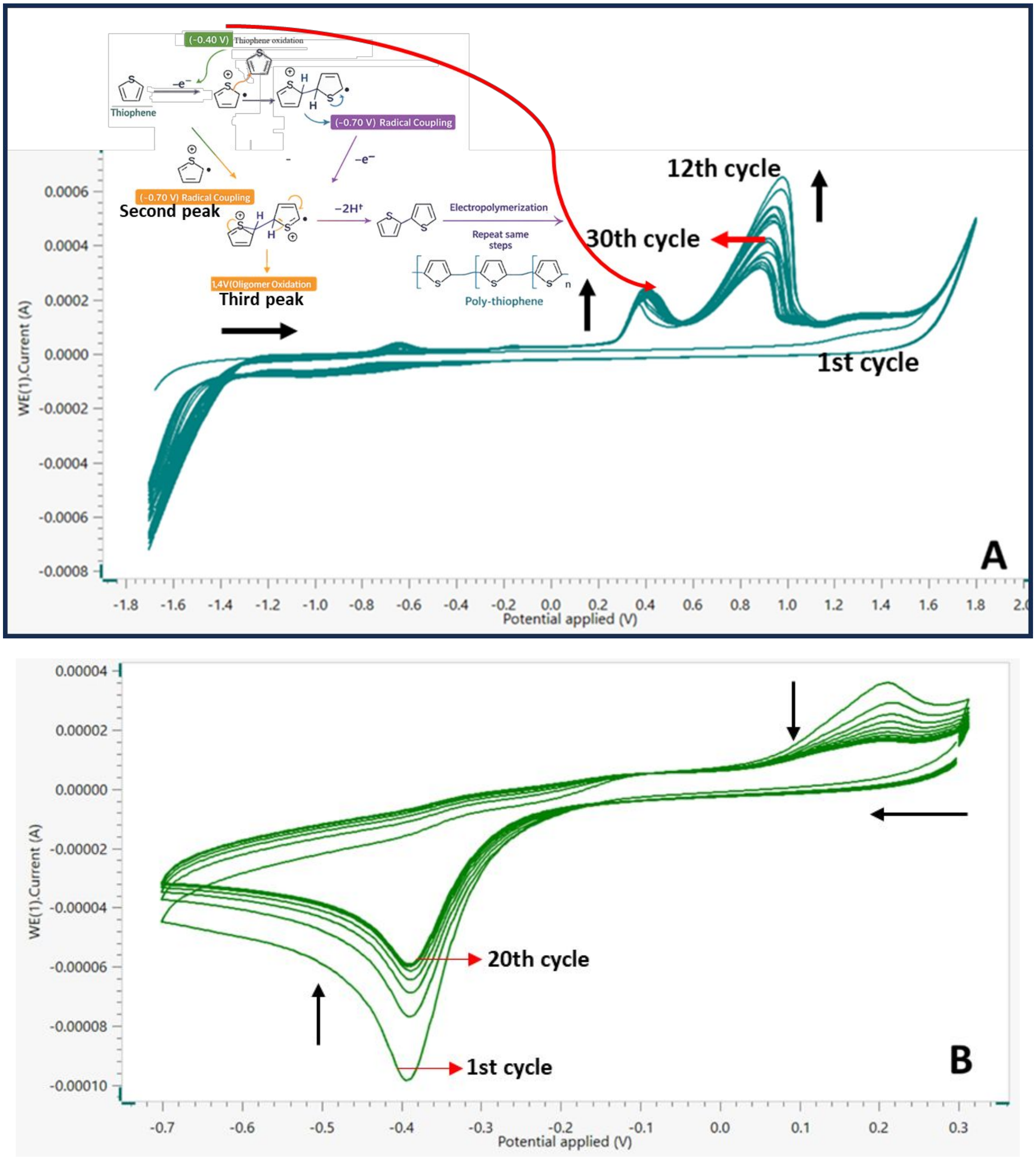

Figure S1. A) CVs of 0.1 M thiophene in $NaClO_4$ + ACN on GCE; B) Cyclic voltammograms Au^{3+} ions in HCl solution on PTh/GCE

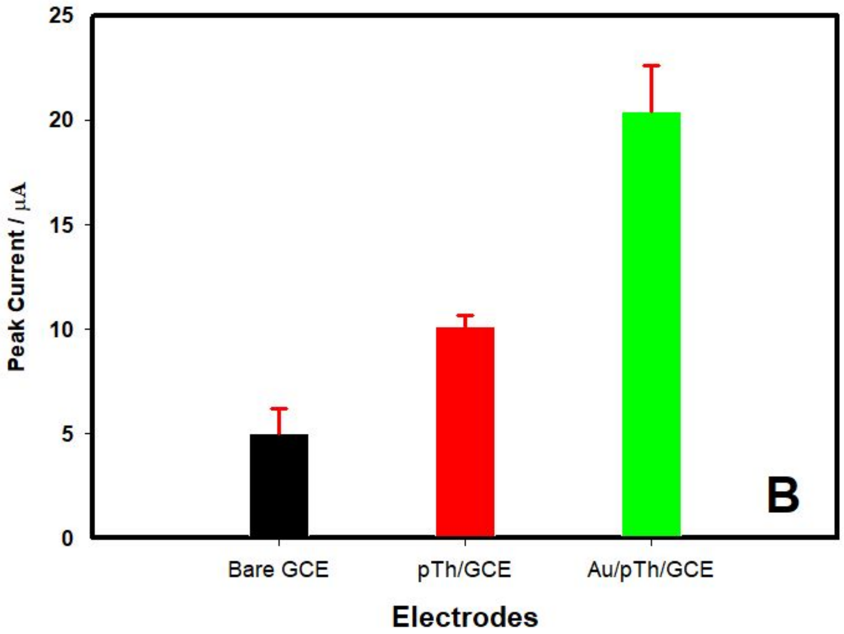

Figure S2 Comparison of peak current responses obtained from different electrodes (bare GCE, pTh/GCE, and Au/pTh/GCE) in the presence of 3.0×10^{-4} mol L^{-1} NLT in pH 6.2 BR buffer. Error bars represent the standard deviation of replicate measurements.

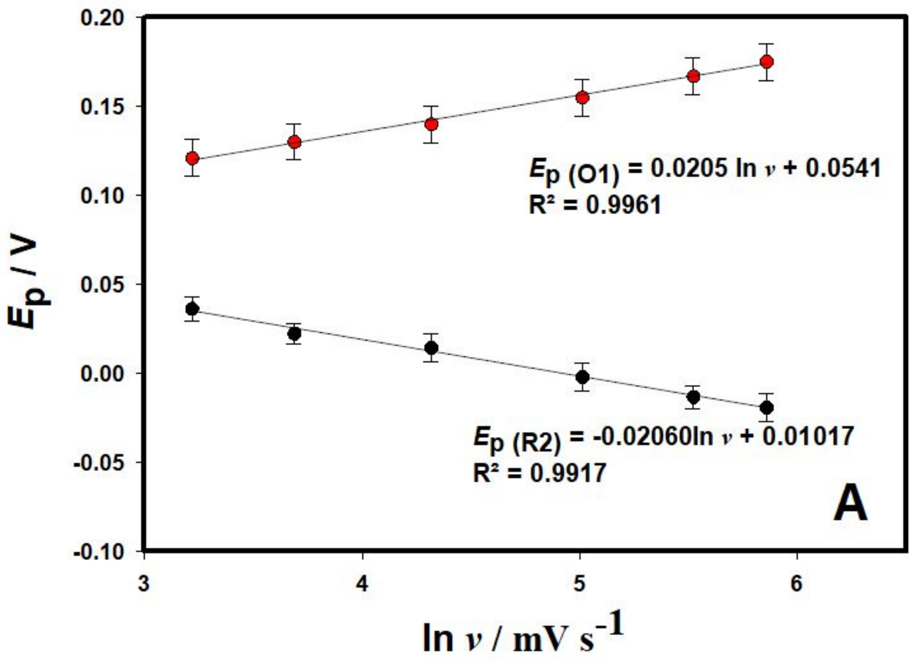

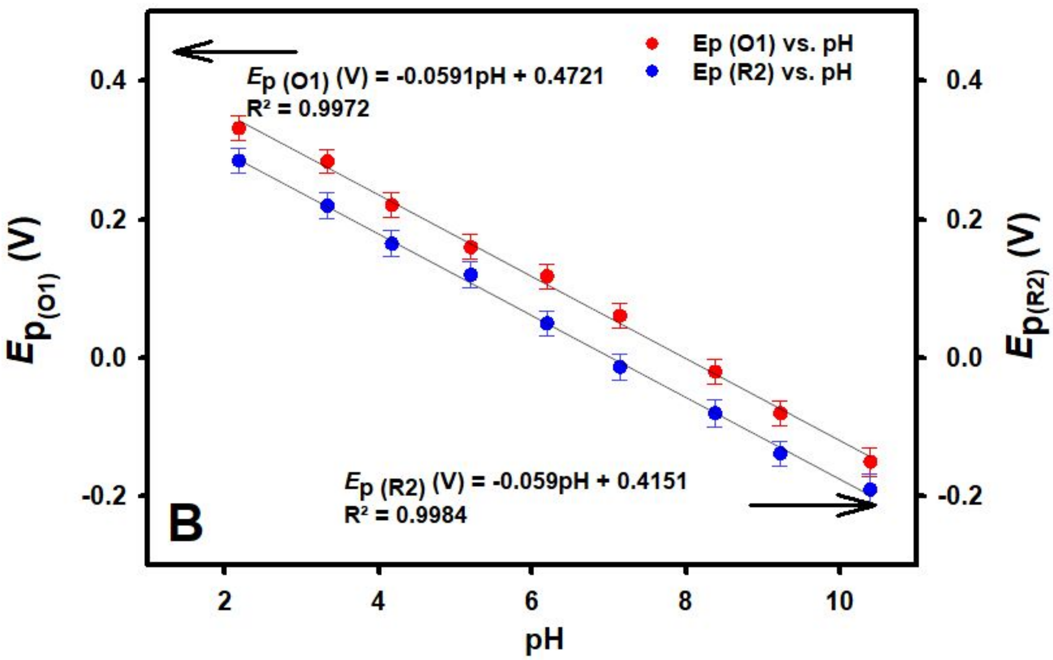

Figure S3. A) lnv-E_{pc} and B) pH-Ep curves for O1/R2 quasi reversible peak in $3.0x10^{-4}$ mol L^{-1} NLT + pH 6.2 BR solution on Au/pTh/GCE

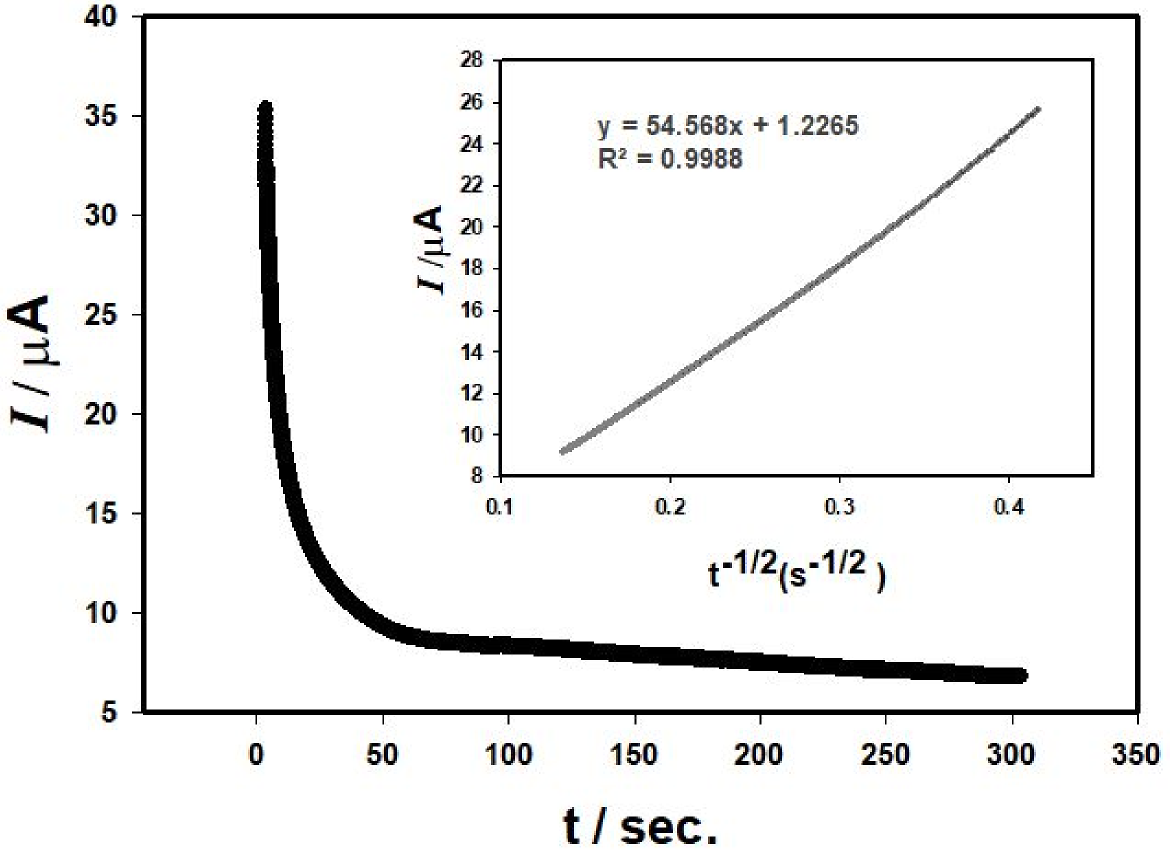

Figure S4. Chronoamperometric curves for NLT obtained at a potential of −0.60 V, inset: the corresponding Cottrell plot ($t^{-1/2}$-I)

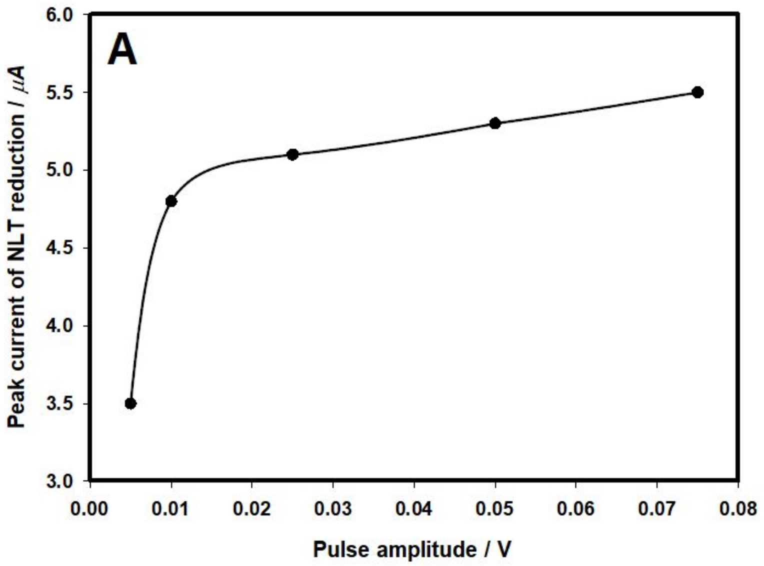

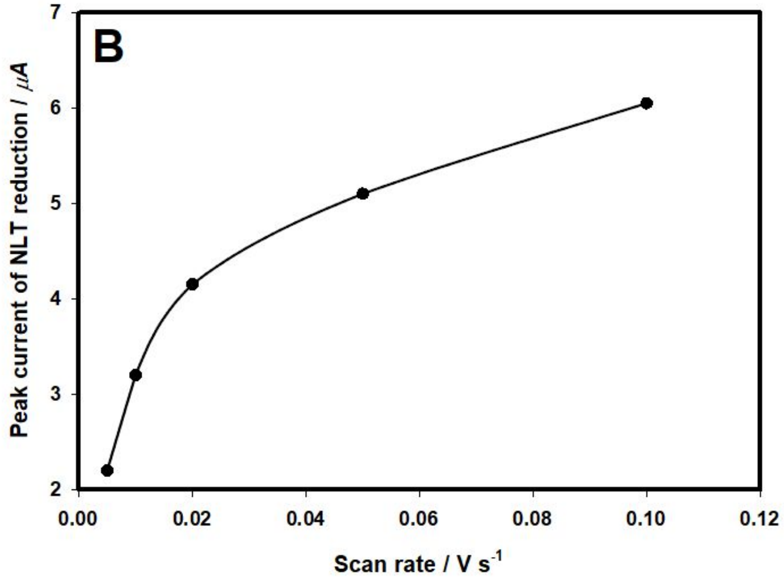

Figure S5. Optimization of differential pulse voltammetry (DPV) parameters for the electrochemical determination of NLT at the Au/pTh/GCE electrode in pH 6.2 BR buffer. (A) Effect of pulse amplitude and (B) effect of scan rate on the reduction peak current of NLT.

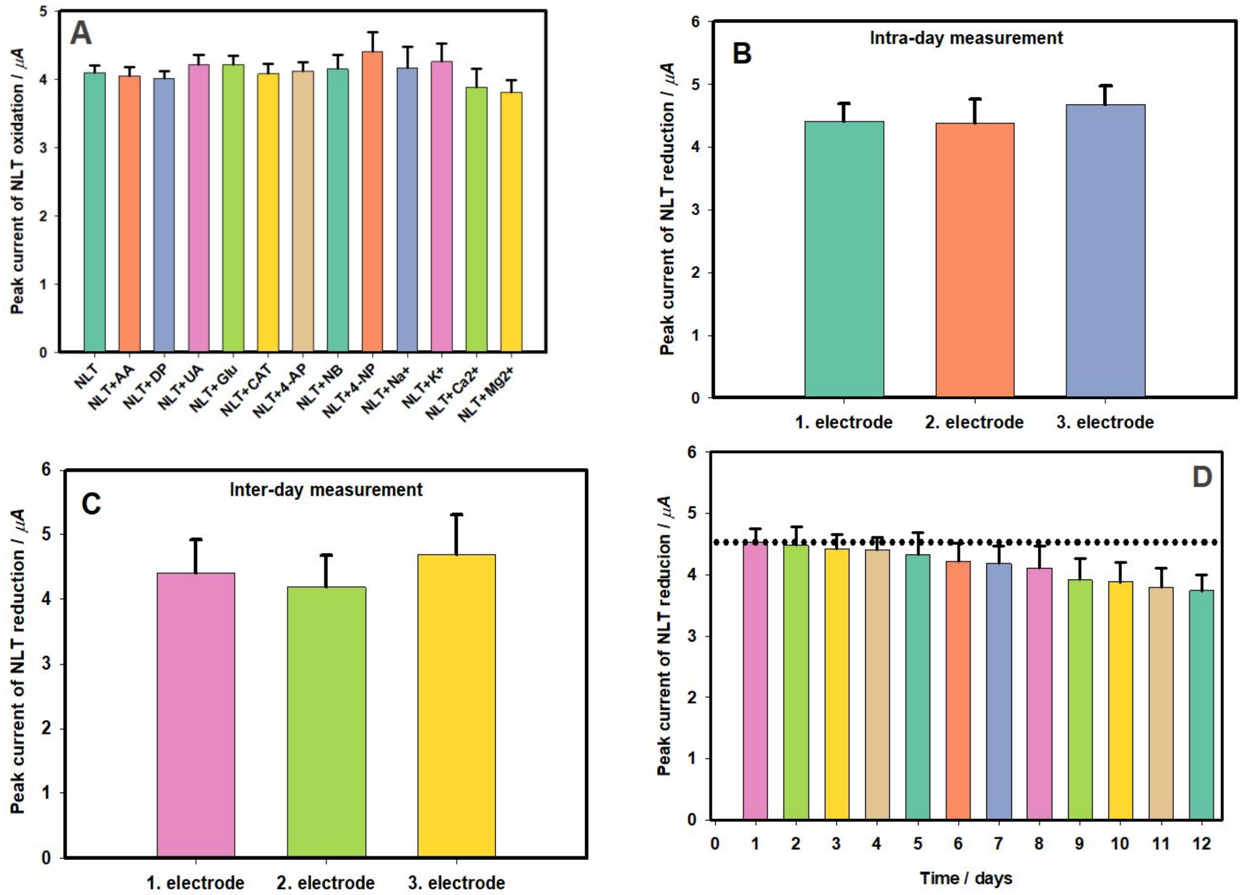

Figure S6. A) Interference studies of the developed Au-PTh/GC electrode for the 2.0 μmol L^{-1} NLT only or with 20.0 μmol L^{-1} AA, DP, UA, Glu, CAT, 4-AP, NB, NP, Na^{+}, K^{+}, Ca^{2+}, Mg^{2+} respectively; reproducibility studies B) intraday-measurements and C) inter-day measurement, D) long term stability studies for 2.0 μmol L^{-1} NLT in pH 6.2 BR buffer.
